# Hydatid thrombosis of the portal vein: a rare case report

**DOI:** 10.1093/jscr/rjaf337

**Published:** 2025-05-23

**Authors:** Fatima Chait, Nourrelhouda Bahlouli, Kaouthar Sfar, Ihssane Lahlou, Meryem Edderai, Jamal El Fenni

**Affiliations:** Radiology Department, Military Teaching Hospital Mohammed V, Mohammed V University, Rabat 10045, Morocco; Radiology Department, Military Teaching Hospital Mohammed V, Mohammed V University, Rabat 10045, Morocco; Radiology Department, Military Teaching Hospital Mohammed V, Mohammed V University, Rabat 10045, Morocco; Radiology Department, Military Teaching Hospital Mohammed V, Mohammed V University, Rabat 10045, Morocco; Radiology Department, Military Teaching Hospital Mohammed V, Mohammed V University, Rabat 10045, Morocco; Radiology Department, Military Teaching Hospital Mohammed V, Mohammed V University, Rabat 10045, Morocco

**Keywords:** portal hydatidosis, cavernoma

## Abstract

Hydatidosis, caused by *Echinococcus granulosus*, is a widespread parasitic disease, especially in livestock areas. In Morocco, it is hyperendemic and poses a major health concern. The liver and lungs are the most affected organs, but portal vein involvement is extremely rare, with only eight reported cases. We present a 56-year-old man with a recurrent hepatic hydatid cyst, three years after a left hepatectomy. Imaging revealed hydatid thrombosis of the portal vein, a rare complication requiring distinct management from typical venous thrombosis.

## Introduction

Hydatid cysts, caused by *Echinococcus granulosus*, remain a public health issue despite medical advances. The liver is the most affected organ (65%–75% of cases) as it serves as the primary barrier against larvae, followed by the lungs (10%–25%). The most common complication is cysto-biliary communication [1]. Another serious complication is free abdominal perforation, which can cause an anaphylactic shock. Here, we present a case of hydatidosis with an atypical site: portal hydatidosis.

## Case report

We report the case of a 56-year-old man who had a history of dog contact during childhood and a prior left hepatectomy for a hepatic cyst, referred to us for follow-up of a recurrent hepatic cyst discovered during a routine ultrasound check-up. The patient had no history of pain, fever, jaundice, nausea, or vomiting. Physical examination was unremarkable. Liver function tests showed a moderate elevation in the ASAT and ALAT levels, which are markers of hepatic cytolysis, without signs of cholestasis.

**Figure 1:**
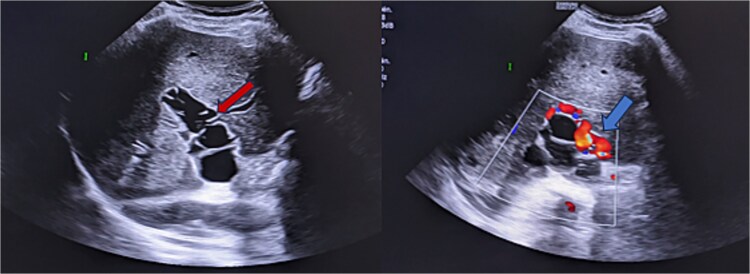
Ultrasound images reveal the presence of multiple liquid cystic formations that do not respond to colour Doppler in the lumen of the portal trunk and its dividing branches. These findings are in relation to portal hydatidosis, with the development of a portal cavernoma.

**Figure 2:**
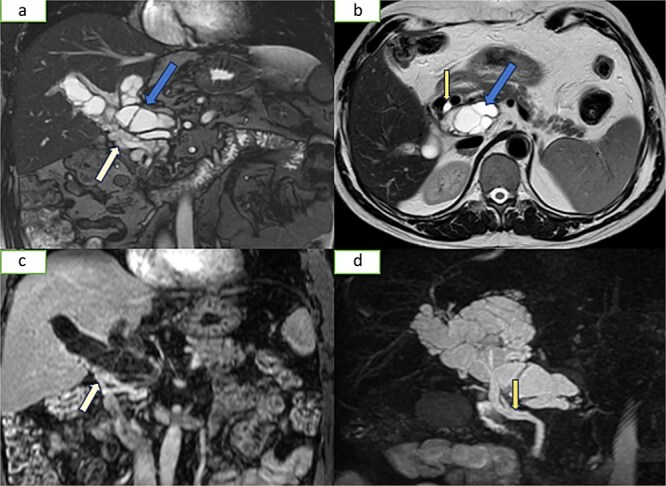
Hepatic MRI images [(a) coronal T2 sequence, (b) axial T2 sequence, (c) coronal T1 injected sequence, (d) 3DBili sequence] show the presence of multiple cysts in the lumen of the portal trunk and its dividing branches. There is also evidence of a portal cavernoma without dilatation of the bile ducts.

**Figure 3:**
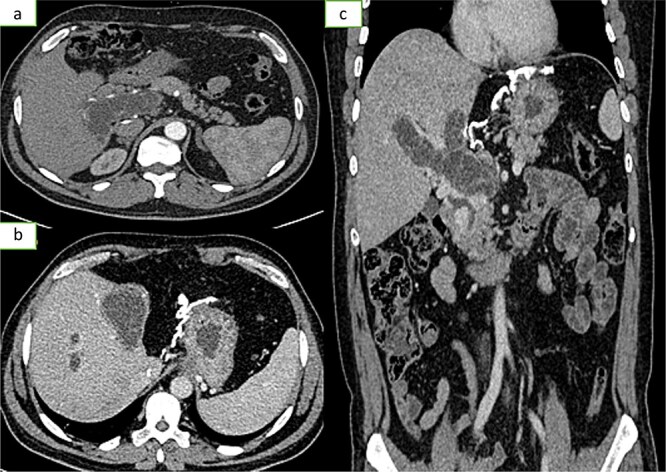
Axial sections of an injected abdominopelvic CT scan [(a) arterial temp; (b) portal temp] and (c) coronal reconstruction showing portal hydatidosis with the presence of a cavernoma of the hepatic hilum.

**Figure 4:**
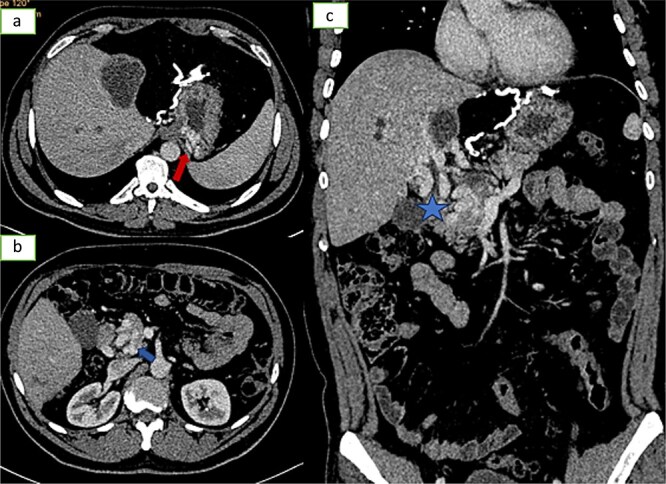
Axial sections (a and b) and coronal reconstruction (c) of an injected abdominopelvic scan showing signs of portal hypertension: gastric varices, peripancreatic varices.

An abdominal ultrasound was performed, which showed multiple cystic lesions within the portal vein, extending to all its anterior and posterior division branches, with venous collaterals at the hepatic hilum, suggesting a portal cavernoma (Fig. 1). A computed tomography (CT) scan and magnetic resonance imaging (MRI) were subsequently conducted for further evaluation, revealing signs of previous left hepatectomy with a multivesicular cyst at the resection margin (stage IIIB) and another in segment V below the liver. The imaging confirmed the presence of multiple hepatic cysts within the portal system, sparing the superior mesenteric vein, spleno-mesenteric trunk, and biliary system (Fig. 2), along with serpiginous collateral venous channels around the hepatic hilum, the pancreatic head consistent with a portal cavernoma and a dilatation of the left gastric wall causing varices of the posterior gastric wall (Fig. 4). Indicating a very rare case of intraportal hydatidosis complicated by signs of portal hypertension (Figs 1–4). Following a thorough review at a multidisciplinary consultation meeting, the surgical option was decisively rejected. This was due to the significant risks of pulmonary embolism due to parasites and thrombotic complications in the portal system or its bypass routes. These risks were deemed too high, especially given the uncertain benefits of surgery, considering the chronic nature of the obstruction, which had been supplanted by the portal cavernoma, and the asymptomatic nature of portal hypertension. As a result, the decision was made to opt for regular monitoring with medical treatment based on albendazole and anticoagulants.

## Discussion

Hydatid cyst disease is a zoonosis caused by *Echinococcus granulosus*, affecting various organs in the body, with a particular predilection for the liver and lungs. The world’s primary hotspots include the Mediterranean basin—especially North Africa—South America, Australia, certain regions of East Africa (notably Kenya), Central Asia, and Northern China [2]. In Morocco, it represents a significant public health problem, where it is considered hyper-endemic.

Human contamination occurs through contact with infected dogs, ingestion of contaminated food or water, and exposure to contaminated soil.

Infestation starts in the gastrointestinal tract, spreading via the portal vein or lymphatics to the liver, where most larvae form cysts. Despite all *Echinococcus* passing through the portal vein, its invasion is rare, likely due to high portal pressure [3].

Portal involvement occurs through several mechanisms: (i) extrinsic compression; (ii) formation of a cyst-portal fistula, allowing daughter cysts to enter the vascular lumen; (iii) cyst rupture, which occurs in 50%–90% of cases; (iv) direct invasion during the migration of parasites through the portal system; (v) clinical presentation of portal hydatidosis, which may include right upper quadrant pain; (vi) signs of portal hypertension, such as hematemesis, ascites, and splenomegaly. Diagnosis of this condition is primarily imaging-based, with no further tests usually required. Ultrasound is typically the first-line modality, revealing dilation of the portal system and the presence of anechoic, uni- or multilocular cystic structures that are not visualized on color Doppler. MRI is the imaging modality of choice for better lesion characterization, exclusion of biliary involvement, and detection of signs of portal hypertension [4, 5].

The main differential diagnoses for portal hydatidosis in imaging are:

1. **Hepatic abscesses**: Absence of membrane and irregular wall, unlike well-defined hydatid cysts.
2. **Hepatic tumours (primary or secondary)**: Irregular lesions with hypervascularization, distinct from smooth hydatid cysts.
3. **Biliary cysts**: Lack of solid wall and internal structures like hydatid vesicles.
4. **Simple liver cysts**: Anechoic lesions without membrane or septa, unlike hydatid cysts.
5. **Chronic hepatitis or cirrhosis**: Changes in liver texture without the typical features of hydatid cysts.
6. **Cystadenomas or cystadenocarcinomas**: Irregular internal septations, in contrast to hydatid vesicles.

Individuals diagnosed with portal hydatidosis necessitate a multidisciplinary treatment strategy to manage both hydatid cysts and the associated portal hypertension.

The medical treatment plan involves the administration of anticoagulants to prevent the formation of blood clots and albendazole, a medication used to treat parasitic infections.

The surgical procedure must be meticulously prepared and planned, with rigorous precautions to be taken during the vascular and anaesthetic phases, as well as during any manual manipulation of the cyst. It should be performed exclusively by centres specializing in liver and vascular surgery, given the significant intraoperative mortality risk. Intraoperatively, once the cystic contents have been evacuated, there is a sharp decrease in pressure and mobilization of the proligeral membrane, leaving the breach uncovered, which can result in intracystic haemorrhage. Thrombosis and a sudden drop in blood pressure have been reported in the literature. Some surgical teams recommend the perioperative use of albendazole to prevent the spread and recurrence of hydatid disease [6, 7].

Previous cases from endemic areas such as Turkey, Spain, Greece, and Chile were treated differently. In Turkey, treatment consisted of surgery in one case, while albendazole was used in another. In Spain and Chile, albendazole was the main treatment, usually for smaller or uncomplicated cysts. In Greece, endoscopic retrograde cholangiopancreatography was used in cases of biliary involvement, highlighting a more specialized approach to complications such as biliary fistulas. These differences illustrate how management strategies are influenced by cyst characteristics, clinical presentation, and treatment options available in different regions [8].

## Conclusion

Hydatid disease remains common and benign in endemic areas, but its rare vascular complications make it serious. Hydatidosis is an exceptionally rare complication. Imaging is essential for diagnosis, mapping vasculature and lesions. Treatment requires a multidisciplinary approach to manage both hydatidosis and portal hypertension.
